# Mirror training device improves dental students’ performance on virtual simulation dental training system

**DOI:** 10.1186/s12909-023-04300-6

**Published:** 2023-05-06

**Authors:** Fengqing Chu, Jue Zheng, Qirui Wang, Xiaoqing Lu, Yue Chen, Yi Zhong, Yingyi Li, Jiali Shi, Yue Jiang, Wei Zhang, Laikui Liu, Wen Sun

**Affiliations:** 1 Department of Basic Science of Stomatology, Affiliated Hospital of Stomatology, Nanjing Medical University, Nanjing, 210029 China; 2 Jiangsu Province Key Laboratory of Oral Diseases, Nanjing, China; 3 Jiangsu Province Engineering Research Center of Stomatological Translational Medicine, Nanjing, China; 4 Nanjing Medical University, Nanjing, China

**Keywords:** Dental students, Dental mirror, Hand skill, Indirect vision, Mirror training, SIMODONT system, Virtual simulation dental training system

## Abstract

**Introduction:**

Clinical practice of dentistry entails the use of indirect vision using a dental mirror. The Mirrosistant is a device that helps dental students become proficient with use of indirect vision mirror operation. This study aimed to explore the role of the Mirrosistant on students’ performance with the virtual simulation dental training system.

**Materials and methods:**

A total of 72 dental students were equally assigned to the Control group and the Experimental group. Subsequently, Mirrosistant was used to conduct a series of mirror training exercises in the Experimental group. The training consisted of tracing the edge and filling in the blank of the prescribed shape, as well as preparing the specified figure on raw eggs using indirect vision via Mirrosistant. Next, both groups were examined using the SIMODONT system, a virtual reality dental trainer, for mirror operation. In addition, a five-point Likert scale questionnaire was used to assess student feedback by using Mirrosistant.

**Results:**

The mirror operation examination conducted by the SIMODONT system revealed that mirror training using Mirrosistant had statistically improved students’ performances (score: 80.42 ± 6.43 vs. 69.89 ± 15.98, *P* = 0.0005) and shorten their performance time of mirror operation (time of seconds: 243.28 ± 132.83 vs. 328.53 ± 111.89, *P* = 0.0013). Furthermore, the questionnaire survey indicated that the participants had positive attitudes toward the mirror training using Mirrosistant. Most students believed that the mirror training device could improve their perceptions of direction and distance, as well as their sensations of dental operation and dental fulcrum.

**Conclusion:**

Mirror training using Mirrosistant can enhance dental students’ mirror perceptual and operational skills on virtual simulation dental training system.

**Supplementary Information:**

The online version contains supplementary material available at 10.1186/s12909-023-04300-6.

## Introduction

Restorative dental treatment is a complex task involving various procedures which require the development and integration of theoretical knowledge, fine motor skills, hand–eye coordination, and spatial perception [1–4]. In order to perform dental procedures, a dentist must be able to work with precision on an extremely small scale. This requires exceptional hand–eye coordination to ensure the safety of patients and proficiency in many different instruments [5, 6].

At present, most dental schools in China adopt a three-stage dental education system of “basic course-preclinical course-clinical practice” to educate dentists [7, 8]. Hand skill course is a required course in dental students’ basic curriculum [5, 9, 10]. It offers students a systematic training approach for hand skills [11–13]. Mirror training is an essential part of hand skill course. A dental mirror enables dentists to view the teeth from all angles, facilitating the detection of any abnormalities. The operation using indirect vision through a dental mirror requires more complex skills. In order to develop mirror skills, perceptual learning and practice are needed to help dental students acquire muscle memory [1, 14]. Effective practice must mimic the same orientation and visual reference points in an actual dental procedure.

In early 1990s, the researchers have been paying attention to mirror training for dental students. Subsequently, Günter et al. developed a training device called Mirroprep (University of Tübingen, Tübingen, Germany) to assist dental students in transferring indirect vision into mirror-inverted movements [15]. However, students’ improvements in performing actual procedures following the training sessions were not reported. Later, Alexander et al. reported an interesting mirror training device named Jumpstart Mirror Trainer (Jumpstart Dental Education, LLC, Fairfax, VA, USA), which is comprised of a rotatable jaw, handpiece-shaped pencil, and multiple interchangeable arches containing activities that mimic dental procedures [16]. Nevertheless, this device is relatively complex and not suitable for large-scale in-class practice in China. Therefore, our study seeks to explore a simple mirror training device and further examine dental students’ performance of mirror operation using virtual reality dental trainer.

In this study, a dental mirror training device, Mirrosistant (Xuanyu, Shanghai, China), was established and tested in a hand skill course at Nanjing Medical University, China. A virtual reality dental trainer, SIMODONT dental trainer (MOOG, NieuwVennep, The Netherlands) system, was used to evaluate the performance of students using indirect vision. In addition, a five-point Likert scale questionnaire was used to assess students’ feedbacks by using Mirrosistant. The purpose of this study is to determine whether the training provided by the Mirrosistant is effective in enhancing students’ mirror perceptual and operational skills.

## Materials and methods

### Participants

A total of 72 dental students in Nanjing Medical University were selected as study subjects, including 30 males and 42 females. All students were in their second year and had no previous dental training. Detailed written informed consent was obtained from all subjects in accordance with protocols approved by the Independent Ethics Committee of the University (permit no: PJ2019-039–001).

### Study design

**Fig. 1:**
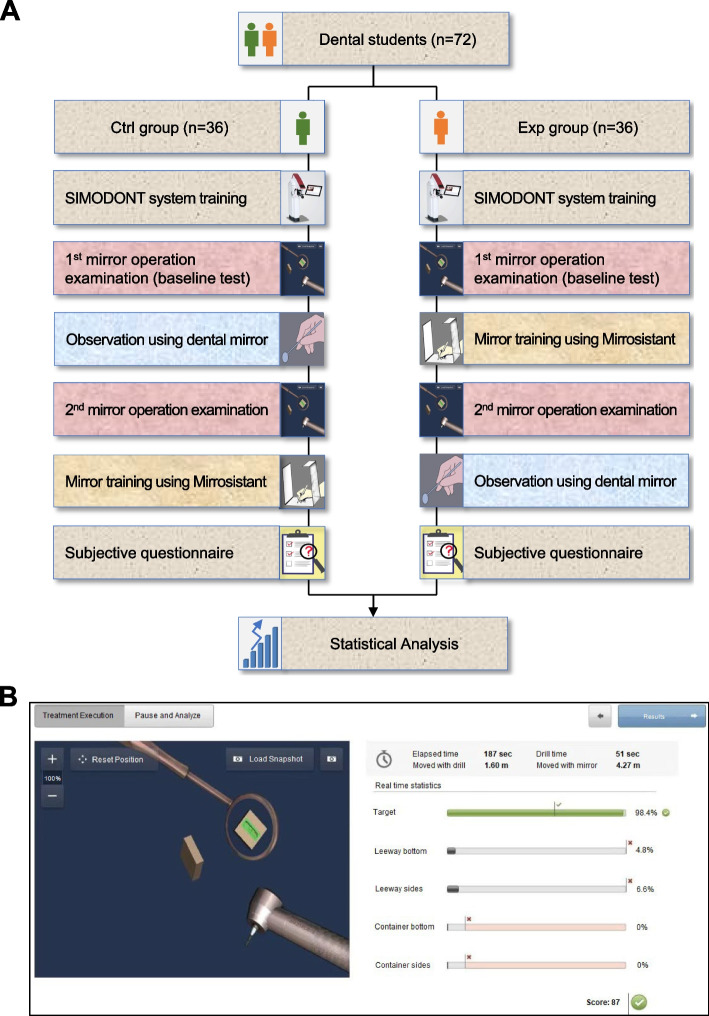
Study methodology and SIMODONT system. **A** Schematic diagram of workflow in this study. **B** Screen snapshot of the SIMODONT system during the mirror operation examination

Seventy-two dental students were randomly divided into two groups: the Control group (Ctrl group, *n* = 36) and the Experimental group (Exp group, *n* = 36). Both groups were subjected to SIMODONT system training (SIMODONT, Japan) and the 1^st^ mirror operation examination was then performed. Afterwards, students were given dentognathic models with complete dentitions and a dental mirror in the Ctrl group. These students were asked to observe a dentognathic model using indirect vision through a dental mirror. In the Exp group, students were given mirror training using Mirrosistant (details showing below). Next, the 2^nd^ mirror operation examination was conducted in both groups. Furthermore, mirror training using Mirrosistant was also given to the Ctrl group to ensure educational equity. Finally, all students were required to complete a subjective questionnaire about the feedbacks on mirror training using Mirrosistant (Fig. 1A).

### SIMODONT system training

Because none of the participants had any experience using SIMODONT system, pre-training was conducted for every participant. Training for the SIMODONT system consisted of basic functions, handpiece grip, dental mirror, fulcrum position, operation procedures, scoring system, and related theories of the use of indirect vision. Each dental student was required to practice for 30 min using direct vision with the SIMODONT system.

### Mirror operation examination

For mirror operation examination, the following task was required to be accomplished within 10 min using SIMODONT system. Each student was asked to prepare a specified rectangle hole with rounded corners by a high-speed handpiece using indirect vision through a dental mirror. This specified rectangle hole was on the back of a cuboid, which was pre-colored with red pixel. The prepared hole was pre-colored with green pixels (Fig. 1B). The SIMODONT system recorded the entire operation process, including total performance time (total time), handpiece working time (preparation time), completion degree of the rectangle hole (target), and edge damage caused by inaccurate operation (leeway bottom, leeway sides, container bottom, container sides). Operation continued until the red portion of the hole had been drilled. The SIMODONT system evaluates student work using a set of standard criteria [17]. The computer measures the percentages of hole that has been removed and over-drilled edge on each side of the hole. Operation score was determined and recorded by the SIMODONT system automatically.

### Mirror training using Mirrosistant

**Fig. 2:**
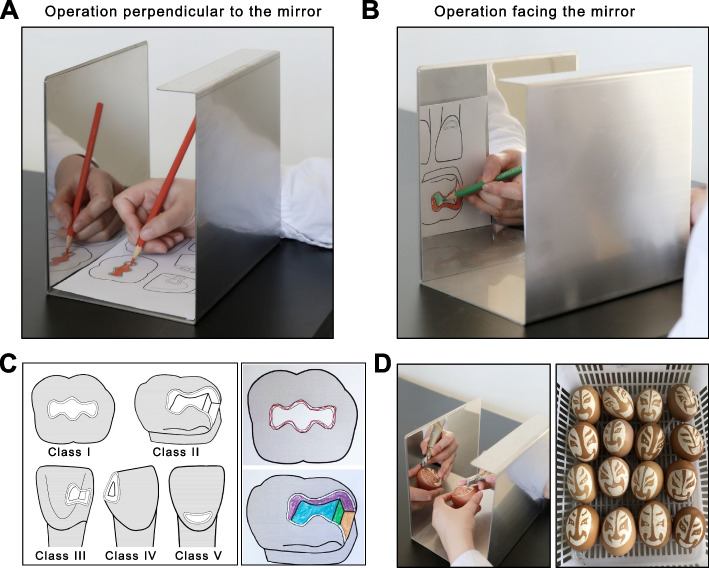
Mirror training using Mirrosistant. **A-B** Representative images of student’s operation using Mirrosistant. **A** the operation perpendicular to the mirror using indirect vision, and **B** the operation facing the mirror using indirect vision. **C** Training session 1. Left panel: test sheet consisting of pictures of G. V. Black’s classification of cavities. Right panel: typical samples of Training session 1 showing how to trace the edge (upper) and fill the blank (lower). **D** Training session 2. Left panel: representative images of student’s operation showing how to prepare the specified figure on the raw egg with Mirrosistant. Right panel: student’s work after training session 2

For mirror training, a mirror training device with modifications was established as previously described [15]. In brief, this mirror training device called Mirroprep (University of Tübingen, Tübingen, Germany), composed of a U-profile (U shaped cross section) high-quality steel sheet with a mirror mounted to its rearmost wall [15]. Owing to its screen, the object positioned on its base plate can be seen only by using a mirror. However, Mirroprep only supports two-dimensional motor skills with indirect vision view. Therefore, we redesigned this mirror training device with a higher rearmost wall to make the object positioned on the screen seen through the mirror of the higher rearmost wall. This may help the operation using indirect vision facing a mirror. This update enables the mirror training device to conduct three-dimensional movements. We named this redesigned mirror training device Mirrosistant (Fig. 2 A & B).

In session 1 of the mirror training using Mirrosistant, the students were required to trace the edge and fill in the blank of the prescribed shapes using indirect vision. The prescribed shapes are G. V. Black’s classification of cavities. The edge tracing may improve the sense of orientation using indirect vision, while the color painting simulates the cavity preparation (Fig. 2C). The students were asked to complete all the drawings within 60 min.

In session 2 of the mirror training using Mirrosistant, the students were required to prepare the specified figure using a handpiece on raw eggs under indirect vision via the mirror. The prepared tracing should have an appropriate depth. If the depth is too shallow, only a portion of the egg shell would be removed. If the tracing is too deep, the egg shell may be perforated and the egg white would leak out (Fig. 2D). The students were asked to complete the exercise within 60 min.

### Questionnaire survey

The survey containing 6 questions were tailored for 72 dental students. An online five-point Likert scale questionnaire was used to assess the feedbacks on mirror training using Mirrosistant (1 = strongly disagree, 2 = somewhat disagree, 3 = neither agree nor disagree, 4 = somewhat agree, and 5 = strongly agree). The survey was administered to the students anonymously.

### Statistical analysis

The efficacy of the mirror training using Mirrosistant was evaluated by analyzing students’ scores on mirror operation examination and questionnaire surveys based on the five-point Likert scale. We tested continuous variables for normal distribution using the Kolmogorov–Smirnov test. Depending on the normality and equality of variances, comparisons between 2 groups were analyzed with 2-tailed unpaired Student’s t-test, unpaired t-test with Welch’s correction or Mann–Whitney test. Comparisons between the 1^st^ and 2^nd^ scores in the same group were analyzed with the paired t-test or the Wilcoxon rank-sum test. Instrument reliability was evaluated by Kendall’s tau *B* and Cronbach’s alpha. Statistical analysis was performed using GraphPad Prism 8 software (GraphPad Software Inc, San Diego, CA, USA). *P* values less than 0.05 were considered statistically significant.

## Results

**Fig. 3:**
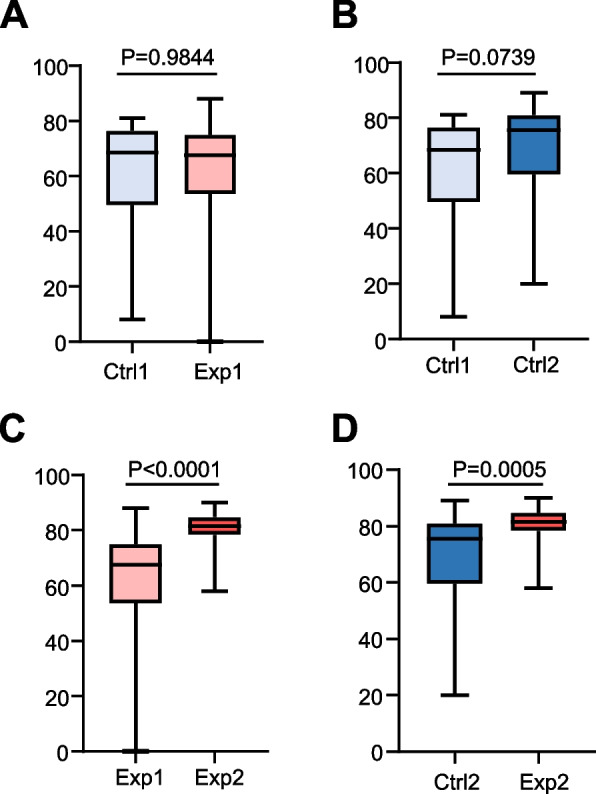
Mirror training using Mirrosistant improves the operation performance of dental students on SIMODONT system. The box plots showing test scores of mirror operation using SIMODONT system. **A** Comparison of the 1^st^ examination between the Ctrl group and the Exp group. The Mann–Whitney test was performed. **B** Comparison of the 1^st^ and 2^nd^ examinations in the Ctrl group. The paired t-test was performed. **C** Comparison of the 1^st^ and 2^nd^ examination in the Exp group. The Wilcoxon rank-sum test was performed. **D** Comparison of the 2^nd^ examination between the Ctrl group and the Exp group. The Mann–Whitney test was performed

Based on the results of the 1^st^ mirror operation examination using SIMODONT system, scores of cavity preparation of the Ctrl and Exp groups were 62.08 ± 17.82 and 61.53 ± 20.61, respectively, with no significant difference between them (*P* > 0.05, Fig. 3A). It is noteworthy that the high value of SDs reflects the presence of confounders such as variation in the hand skills between participants. Subsequently, Mirrosistant was used to adopt a series of mirror training among the students in the Exp group.

To determine the role of mirror training using Mirrosistant, the 2^nd^ mirror operation examination using the SIMODONT system was conducted. The results showed that the Ctrl group’s score on the 2^nd^ examination was slightly higher than that of the 1^st^ examination (69.89 ± 15.98 vs. 62.08 ± 17.82). However, the difference was not significant (*P* > 0.05, Fig. 3B). In contrast, after mirror training using Mirrosistant, the Exp group’s score on the 2^nd^ examination was statistically higher than that of the 1^st^ examination (80.42 ± 6.43 vs. 61.53 ± 20.61, *P* < 0.0001, Fig. 3C). In addition, the 2^nd^ operational score of the Exp group (80.42 ± 6.43) was statistically higher than that of the Ctrl group (69.89 ± 15.98, *P* = 0.0005, Fig. 3D). Therefore, these data suggest that the mirror training using Mirrosistant improved the performance of dental students on SIMODONT system.

**Fig. 4:**
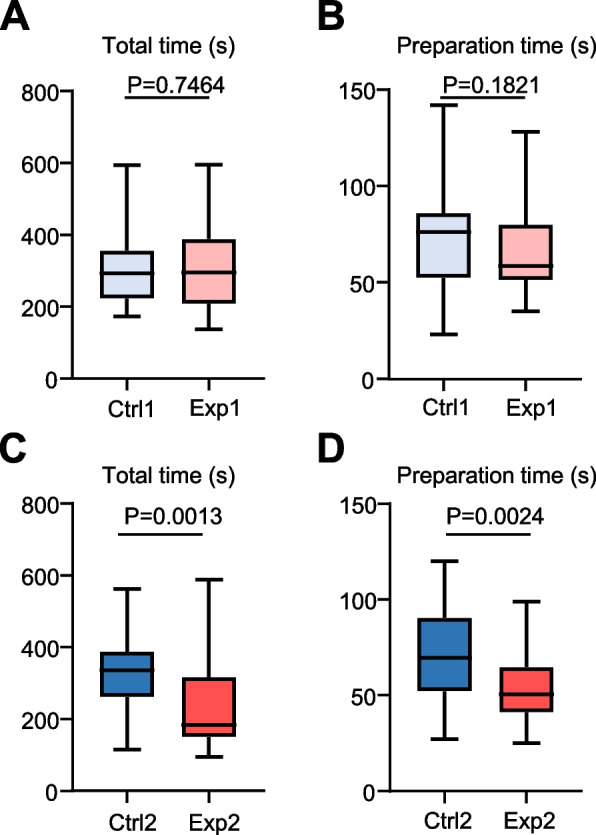
Mirror training using Mirrosistant shortens the performance time of dental students on SIMODONT system. The box plots showing the time of dental students using the SIMODONT system. **A** Total time of seconds taken to complete the mirror operation were compared between the Ctrl group and the Exp group in the 1^st^ examination. A two-tailed unpaired Student’s t-test was performed. **B** Preparation time of seconds during mirror operation were compared between the Ctrl group and the Exp group in the 1^st^ examination. The Mann–Whitney test was performed. **C** Total time of seconds taken to complete the mirror operation were compared between the Ctrl group and the Exp group in the 2^nd^ examination. The Mann–Whitney test was performed. **D** Preparation time of seconds during mirror operation were compared between the Ctrl group and the Exp group in the 2^nd^ examination. The unpaired t-test with Welch’s correction was performed

To determine the effect of mirror training using Mirrosistant on students’ proficiency during mirror operation, we used the SIMODONT system to record the total performance time (total time) and dental handpiece working time (preparation time). First, there was no significant difference between the Exp group and the Ctrl group in terms of total time (time of seconds: 315.72 ± 133.22 vs. 306.81 ± 97.02, *P* > 0.05, Fig. 4A). Moreover, there was no statistical difference between groups in terms of the time spent using the dental handpiece (time of seconds: 65.50 ± 21.47 vs. 72.50 ± 25.21, *P* > 0.05, Fig. 4B). After mirror training using Mirrosistant, the students in the Exp group spent significantly less time than the Ctrl group (time of seconds: 243.28 ± 132.83 vs. 328.53 ± 111.89, *P* = 0.0013, Fig. 4C). In addition, the dental handpiece working time was significantly reduced (time of seconds: 53.86 ± 17.33 vs. 69.97 ± 25.22, *P* = 0.0024, Fig. 4D). Therefore, the data suggest that mirror training using Mirrosistant has significantly reduced the time of dental students’ using the SIMODONT system.

**Fig. 5:**
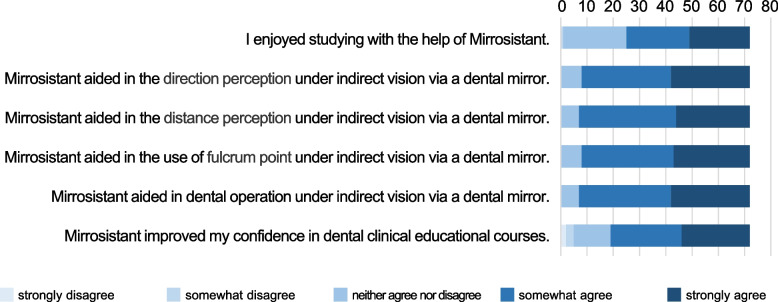
Students’ feedback. A five-point Likert scale questionnaire was used to collect the feedback from dental students (*n* = 72). Each response was rated on a five-point Likert scale. Responses were categorized as positive (somewhat agree, strongly agree), neutral (neither agree nor disagree), and negative responses (somewhat disagree, strongly disagree)

To gain the students’ opinions and feedbacks on the mirror training using Mirrosistant, we conducted a survey among 72 students with a Likert scale questionnaire. The Kendall’s tau B was ranged 0.787 to 0.987 (median, 0.869) and Cronbach’s alpha was 0.974, representing reasonable reliability of the instruments used in this study (Supplementary Table 1). All the questions of the questionnaire and the composition ratios of responses are shown in Fig. 5 and Supplementary Table 2. Generally, the students’ response to the mirror training using Mirrosistant was positive. Most students believed that mirror device training could improve their perceptions of direction and distance, as well as their sensations of dental operation and dental fulcrum.

## Discussion

It is necessary for dentists to use indirect vision skillfully to indirectly observe teeth surfaces, as well as accurately discern direction and distance. Previous studies have shown that the perceptual ability of mirror operation can be obtained and improved [16]. Muscle-memory can be improved through consistent training [18]. Mirror training may shorten the students’ adaptation period to the use of dental mirrors and reduce their reliance on direct vision during dental procedures. Many colleges have developed a variety of methods to train dental students using indirect vision. Such methods include direct use of dental mirror for training, the application of portable dental mirror training equipment, and the training with virtual simulation dental training system [16, 19].

**Fig. 6:**
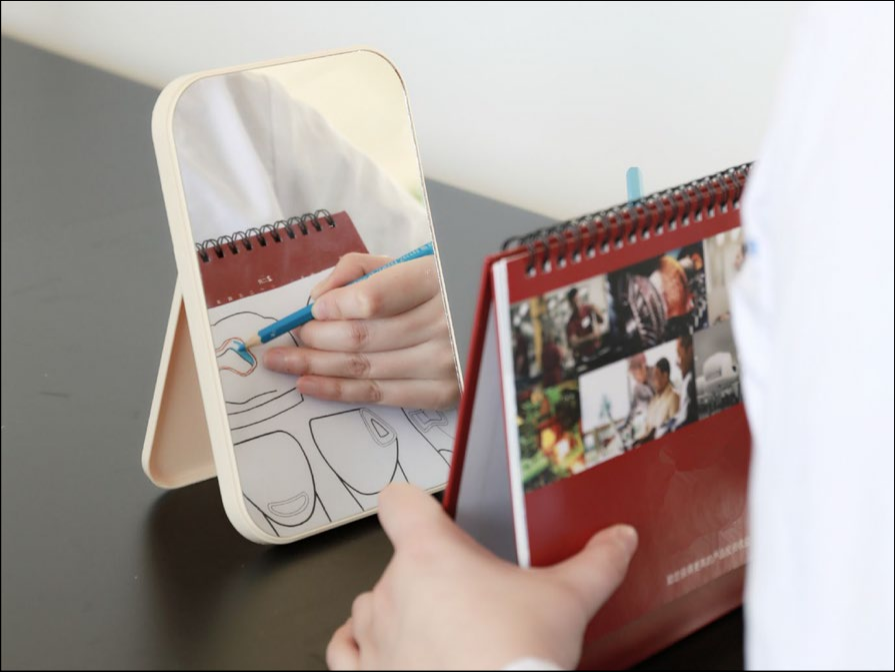
Use materials in daily life to simulate Mirrosistant

In the current study, a simple mirror device called Mirrosistant was established and tested in a hand skill course for dental students. Mirrosistant is easy to operate, easy to install and has low maintenance cost. Importantly, Mirrosistant can be manufactured with easily obtained materials (Fig. 6). It is convenient for students to learn and practice independently. In addition, it has been demonstrated that the mirror training using Mirrosistant can significantly improve students’ perceptual and operational ability. However, it remains unknown whether the skills acquired through such training could be beneficial to clinical practice. Therefore, the mirror training using Mirrosistant should be assessed not only through course examination and questionnaire, but also through the objective evaluation of dental procedures in the near future.

Although mirror training with Mirrosistant has a positive impact on students’ perceptions of direction and distance, the simple mirror training device could not replace virtual simulation dental training system, such as Voxel-Man simulator (Voxel-Man, Hamburg, Germany) [20], Kobra Simulator (Haptikfabriken AB, Stockholm, Sweden) [21], and SIMODONT [22]. Presently, virtual reality simulators are becoming an essential part of modern education [23, 24]. In pre-clinical settings, virtual reality applied in dentistry constantly serves as a method or an adjunct to improve fine motor skills and hand–eye coordination [25, 26]. However, SIMODONT system has some disadvantages, such as initial high installation cost, slow update of software functions, and lack of effective training in communication skills. In our experience, students prefer traditional indirect dental mirror training on plastic teeth and an actual headpiece compared to using the virtual simulation dental training system. Indeed, virtual simulation dental training system does not give a realistic sense of manipulation and it does not mimic the narrow operating space of the mouth.

### Study limitations

Notably, Mirrosistant has only two reflecting angles: the vertical angle and the facing angle, rendering it incapable of simulating additional reflection angles existing in clinical procedures. An additional limitation is that the presence of variation in the hand skills between participants, which may camouflage the actual results. This variation should be controlled in future studies, and we can set up a baseline test to remove some participants with poor hand skills. Also, future studies may investigate different training periods to determine the optimal training period for the training device. Finally, the variation in the intervention between the Ctrl and Exp groups may conceal the actual results.

## Conclusion

In summary, mirror training using Mirrosistant has enhanced dental students’ use of indirect vision and operational skills on a virtual simulation dental training system. Therefore, our study shows that the Mirrosistant training system was perceived by students to be a useful component of the dental training.

## Supplementary Information

**Additional file 1: Supplementary Table 1.** Kendall’s tau B of the questionnaire survey based on the five-point Likert scale. **Supplementary Table 2.** A five-point Likert scale questionnaire was used to collect the feedback from dental students (*n*=72)

## Data Availability

The authors declare that all the data and materials supporting the findings of this study are available within the article and are available from the corresponding author upon request.
