# Supplementary material for: Mirror training device improves dental students’ performance on virtual simulation dental training system
Source: BMC Med Educ. 2023 May 6;23:315. doi: 10.1186/s12909-023-04300-6 (PMC10163732; doi:10.1186/s12909-023-04300-6)
Supplement: Supplementary file 1 — Additional file 1: Supplementary Table 1. Kendall’s tau B of the questionnaire survey based on the five-point Likert scale. Supplementary Table 2. A five-point Likert scale questionnaire was used to collect the feedback from dental students (n=72) [file 12909_2023_4300_MOESM1_ESM.docx]

**Supplementary Table 1. Kendall’s tau B of the questionnaire survey based on the five-point Likert scale**

|  | **Q1** | **Q2** | **Q3** | **Q4** | **Q5** | **Q6** |
| --- | --- | --- | --- | --- | --- | --- |
| **Q1** | 1.00 | 0.79 | 0.797 | 0.795 | 0.787 | 0.88 |
| **Q2** | 0.79 | 1.00 | 0.945 | 0.979 | 0.987 | 0.851 |
| **Q3** | 0.797 | 0.945 | 1.00 | 0.965 | 0.958 | 0.869 |
| **Q4** | 0.795 | 0.979 | 0.965 | 1.00 | 0.966 | 0.861 |
| **Q5** | 0.787 | 0.987 | 0.958 | 0.966 | 1.00 | 0.847 |
| **Q6** | 0.88 | 0.851 | 0.869 | 0.861 | 0.847 | 1.00 |

Q1：I enjoyed studying with the help of Mirrosistant.

Q2：Mirrosistant aided in the direction perception under indirect vision via a dental mirror.

Q3：Mirrosistant aided in the distance perception under indirect vision via a dental mirror.

Q4：Mirrosistant aided in the use of fulcrum point under indirect vision via a dental mirror.

Q5：Mirrosistant aided in dental operation under indirect vision via a dental mirror.

Q6：Mirrosistant improved my confidence in dental clinical educational courses.

**Supplementary Table 2. A five-point Likert scale questionnaire was used to collect the feedback from dental students (n=72)**

|  | Students’ feedback | | | | |  |  |
| --- | --- | --- | --- | --- | --- | --- | --- |
|  | *Strongly agree (%)* | *Somewhat agree (%)* | *Neutral (%)* | *Somewhat disagree (%)* | *Strongly disagree (%)* | *Means* | *SDs* |
| 1. I enjoyed studying with the help of Mirrosistant. | 23 (31.9%) | 24 (33.3%) | 24 (33.3%) | 1 (1.4%) | 0 (0.0%) | 3.81 | 0.91 |
| 1. Mirrosistant aided in the direction perception under indirect vision via a dental mirror. | 30 (41.7%) | 34 (47.2%) | 8 (11.1%) | 0 (0.0%) | 0 (0.0%) | 4.22 | 0.71 |
| 1. Mirrosistant aided in the distance perception under indirect vision via a dental mirror. | 28 (38.9%) | 37 (51.4%) | 7 (9.7%) | 0 (0.0%) | 0 (0.0%) | 4.19 | 0.71 |
| 1. Mirrosistant aided in the use of fulcrum point under indirect vision via a dental mirror. | 29 (40.3%) | 35 (48.6%) | 8 (11.1%) | 0 (0.0%) | 0 (0.0%) | 4.15 | 0.77 |
| 1. Mirrosistant aided in dental operation under indirect vision via a dental mirror. | 30 (41.7%) | 35 (48.6%) | 7 (9.7%) | 0 (0.0%) | 0 (0.0%) | 4.21 | 0.73 |
| 1. Mirrosistant improved my confidence in dental clinical educational courses. | 26 (36.1%) | 27 (37.5%) | 14 (19.4%) | 3 (4.2%) | 2 (2.8%) | 3.92 | 0.97 |
